# Gastrointestinal Stromal Tumor: GIST Another Duodenal Ulcer

**DOI:** 10.31486/toj.18.0167

**Published:** 2020

**Authors:** Patrick S. Harris, John Romano, Kirk B. Russ, Mohamed G. Shoreibah, Kondal Rao Kyanam Kabir Baig

**Affiliations:** ^1^ Tinsley Harrison Internal Medicine Residency, University of Alabama at Birmingham, Birmingham, AL; ^2^ Division of Gastroenterology and Hepatology, Medical University of South Carolina, Charleston, SC; ^3^ Division of Gastroenterology and Hepatology, University of Alabama at Birmingham, Birmingham, AL

**Keywords:** *Duodenal ulcer*, *endoscopy–gastrointestinal*, *gastrointestinal hemorrhage*, *gastrointestinal stromal tumors*

## Abstract

**Background:** Gastrointestinal stromal tumors (GISTs), although exceedingly rare, are the most common mesenchymal tumors in the gastrointestinal (GI) tract. GISTs are often asymptomatic; approximately 10% are found incidentally on imaging or endoscopy for other indications, although GI bleeding, intestinal obstruction, and perforation can occur. We present a case of upper GI bleeding from a duodenal GIST. Proton-pump inhibitor (PPI) therapy resulted in complete endoscopic ulcer healing, yet a discrete mass lesion was identified on endoscopic ultrasound (EUS).

**Case Report:** A 70-year-old female presented with upper GI bleeding, and a duodenal ulcer was identified with esophagogastroduodenoscopy (EGD). Computed tomography (CT) scan of the abdomen and pelvis showed duodenal bulb thickening without clear mass. The ulcer was treated with 1:10,000 concentration epinephrine, injected in 4 quadrants around the ulcer base. The patient's GI bleeding resolved, and she was discharged with a referral for outpatient EUS follow-up. One month later, EUS showed resolution of the ulcer after PPI therapy but also showed a lesion consistent with GIST that was confirmed by cytologic analysis. The patient was started on imatinib therapy and had no further bleeding.

**Conclusion:** Initial EGD and CT findings could have easily been attributed to duodenal peptic ulcer disease for which follow-up endoscopy is not routinely recommended given the low risk of malignancy. However, because of the high index of suspicion on the part of the referring physicians, duodenal GIST was diagnosed. This case extends the spectrum of the presentation, evaluation, and diagnosis of GISTs and stresses the importance of keeping this rare disease on the provider's differential, even after routine workup shows no findings of tumor.

## INTRODUCTION

Gastrointestinal stromal tumors (GISTs), although rare, are the most common mesenchymal tumors in the gastrointestinal (GI) tract and can occur from mouth to anus, with a predilection for the stomach and proximal small intestine.^1^ GISTs are often asymptomatic, with approximately 10% found incidentally on imaging or endoscopy for other indications, although GI bleeding, intestinal obstruction, and perforation can occur.^1^ The estimated overall incidence of GISTs is 11 to 12.7 per million individuals, according to data collected in population-based studies.^2,3^ GISTs show no predilection for sex. While some cases have been reported in children, the majority of cases (90%) occur in patients >40 years, with a median diagnosis age of 63 years.^2,3^

We present a case of upper GI bleed from a duodenal GIST in which complete endoscopic healing was seen, but a discrete mass lesion was identified on endoscopic ultrasound (EUS).

## CASE REPORT

A 70-year-old female with a medical history of thyroidectomy for benign thyroid nodules, hyperlipidemia, and hypertension presented to a local hospital after experiencing several melenic stools and a presyncopal episode. She was found to be anemic with a hemoglobin level of 8.8 g/dL from a baseline of 13 g/dL. Platelets were 257 × 10^9^/L (reference range, 140-400 × 10^9^/L), and international normalized ratio was 1.13 (reference range, <1.10). Other laboratory values were unremarkable. The patient was transfused 4 units of packed red blood cells, started on oral proton-pump inhibitor (PPI) therapy twice daily, and transferred to our institution for further management of the upper GI bleed.

**Figure 1.**
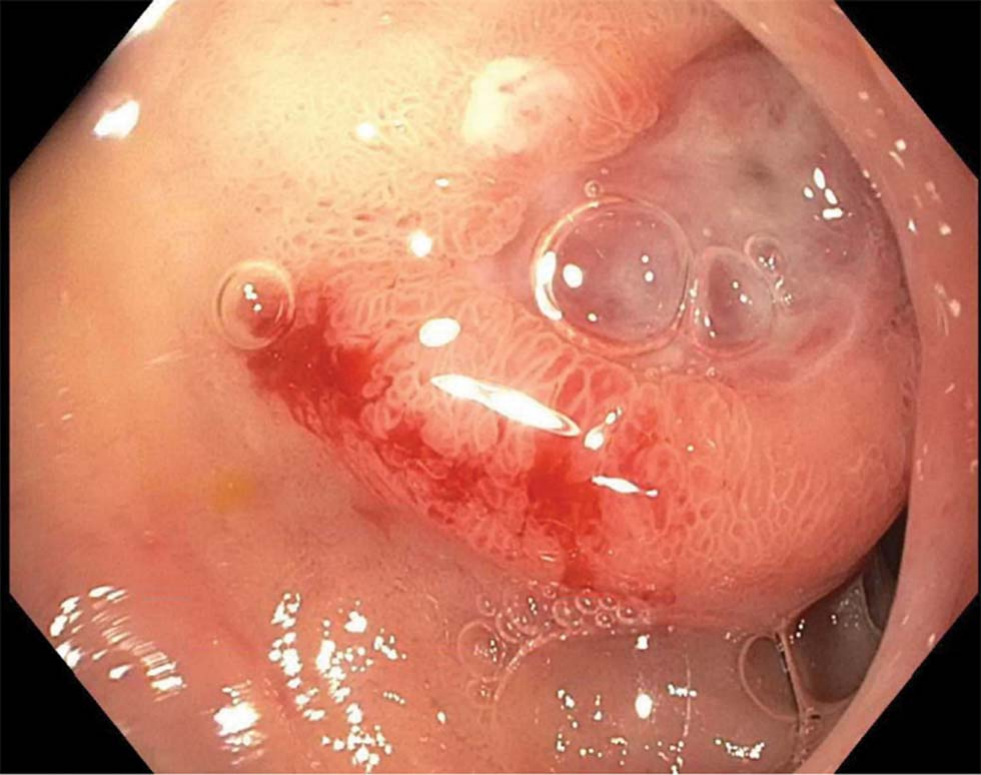
**Initial esophagogastroduodenoscopy revealed a 1-cm, clean based, slightly protuberant ulcer in distal duodenal bulb with surrounding heaped-up edges.**

Esophagogastroduodenoscopy (EGD) displayed a Forrest classification 1B duodenal bulb ulcer (active oozing) with a slightly protuberant appearance, concerning for an underlying mass lesion (Figure 1).^4^ The ulcer was treated with 1:10,000 concentration epinephrine, injected in 4 quadrants around the ulcer base. Computed tomography (CT) scan of the abdomen and pelvis displayed duodenal bulb thickening without clear mass. Immunostain for *Helicobacter pylori* on gastric biopsies was negative, and the patient denied nonsteroidal antiinflammatory drug use. Biopsies from the ulcer margin displayed gastric heterotopia with no evidence of malignancy. The patient's GI bleeding resolved, and she was discharged with continued PPI and referral for outpatient EUS.

**Figure 2.**
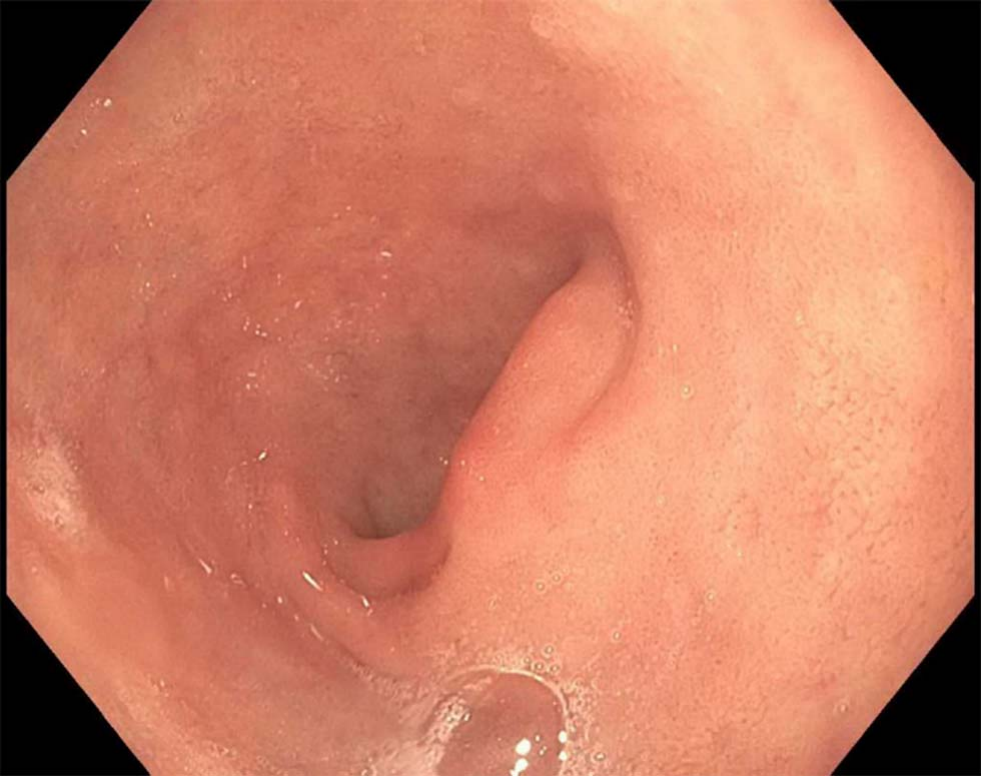
**Duodenal bulb on follow-up esophagogastroduodenoscopy showed healed duodenal ulcer and no evidence of luminal mass.**

**Figure 3.**
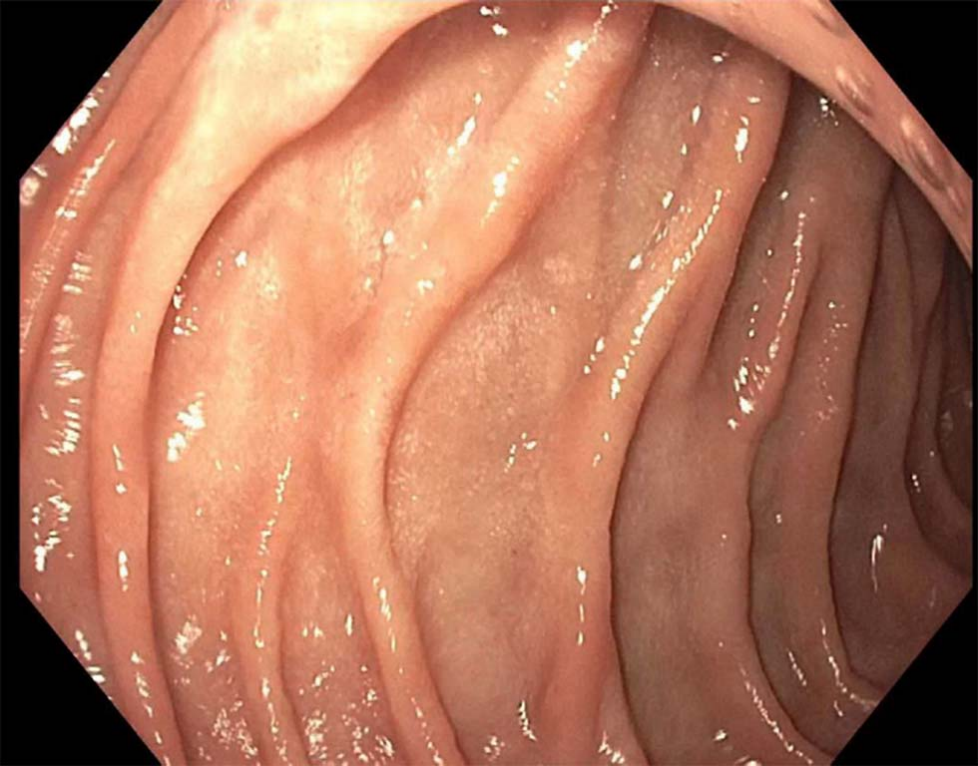
**Duodenal sweep on follow-up esophagogastroduodenoscopy showed healed duodenal ulcer and no evidence of luminal mass.**

**Figure 4.**
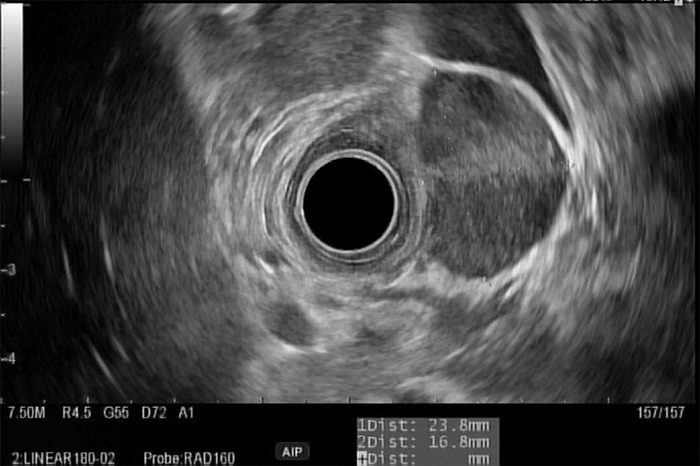
**Endoscopic ultrasound in duodenal bulb showed a 23.8 mm × 16.8 mm submucosal, hypoechoic mass in the muscularis propria.**

One month following discharge, the patient presented to our outpatient endoscopy center for EGD/EUS. Follow-up EGD showed complete endoscopic healing of the previously seen duodenal ulcer and no evidence of intrinsic or submucosal luminal mass (Figures 2 and 3). However, EUS displayed a discrete, hypoechoic, 23.8 mm × 16.8 mm, extrinsic lesion in the duodenal bulb, without evidence of invasion into adjacent structures (Figure 4). Pathologic findings from fine-needle aspiration were consistent with GIST. Cytologic samples were positive for CD117 (C-KIT), negative for S100, and weakly positive for smooth muscle actin, supporting the diagnosis of GIST. CT scans of chest, abdomen, and pelvis showed no evidence of metastatic disease.

The patient was referred to the oncology clinic and was started on neoadjuvant imatinib 400 mg daily. As of the writing of this report, the patient remained on imatinib with acceptable tolerance. Her hemoglobin was stable at 10.9 g/dL, and she had no further GI bleeding. Repeat CT/EUS in the coming months will be used to evaluate her response to therapy and consider surgical resection.

## DISCUSSION

In this case, complete endoscopic healing of an upper GI bleed from a duodenal GIST was obtained with PPI therapy, yet a discrete mass lesion was identified on EUS. Initial EGD and CT findings could have easily been attributed to duodenal peptic ulcer disease for which follow-up endoscopy is not routinely recommended given the low risk of malignancy.^5^ However, because of the high index of suspicion on the part of the referring physicians, duodenal GIST was diagnosed.

The presentation of GIST varies, with symptoms occurring in only 70% of patients and usually attributable to the presence of a mass effect or bleeding.^1^ The most common presenting complaint is GI bleeding, in addition to abdominal pain, nausea/vomiting, or early satiety. A large majority of GISTs are found in the stomach (50% to 60%), followed by the small intestine (30% to 40%), but they have been reported throughout the GI tract and rarely present as extra gastrointestinal tumors in the mesentery or retroperitoneum.^1^ The discovery of gain-of-function mutations in CD117 (C-KIT protein), a tyrosine kinase growth factor receptor present in 85% to 94% of GIST cells, has revolutionized the ability to diagnose GIST tumors.^6^ Other novel markers include smooth muscle actin, expressed in approximately 30% of GISTs, and S100, present in 5% of GISTs.^6^ EUS plays a crucial role in the diagnosis of GISTs as it permits fine-needle aspiration sampling of suspicious lesions and thus the ability to perform the aforementioned cytologic analyses.^7^

Multiple characteristics are associated with malignancy, including tumor size >4 cm, echogenic foci and cystic spaces on endosonographic evaluation, and an irregular extraluminal border.^8^ The management of GISTs depends on the malignant potential, with tumor size, tumor site, and mitotic index serving as valuable predictors of malignant behavior. The preferred treatment for localized GIST is complete surgical resection, with an estimated 5-year survival of 48% to 65%.^9^ Patients with large tumor size, presence of tumor rupture, high mitotic index, and nongastric location have higher risks for recurrence and are thus often treated with adjuvant chemotherapy, most commonly with imatinib, a tyrosine kinase inhibitor. Imatinib is also first-line therapy for tumors that are not amenable to resection, metastatic GISTs, and recurrent GISTs. Second-line therapy with sunitinib, a C-KIT and platelet-derived growth factor receptor alpha kinase inhibitor, has shown promising results.^6^ Recurrence of GISTs is common, with a predilection to recur in the peritoneum or liver, highlighting the importance of long-term follow-up for all patients.^10^

## CONCLUSION

While GISTs remain rare and often asymptomatic, they can be a source of upper GI bleeding and must be considered when alternate etiologies of peptic ulcers are excluded. We present this case to expand the spectrum of the presentation of GISTs, highlight the evaluation and diagnosis of GISTs, and stress the importance of keeping this rare disease in the differential diagnosis of bleeding peptic ulcers.

## Notes

This article meets the Accreditation Council for Graduate Medical Education and the American Board of Medical Specialties Maintenance of Certification competencies for Patient Care and Medical Knowledge.
